# Single Gene Deletions of Orexin, Leptin, Neuropeptide Y, and Ghrelin Do Not Appreciably Alter Food Anticipatory Activity in Mice

**DOI:** 10.1371/journal.pone.0018377

**Published:** 2011-03-28

**Authors:** Keith M. Gunapala, Christian M. Gallardo, Cynthia T. Hsu, Andrew D. Steele

**Affiliations:** Broad Fellows Program in Brain Circuitry, Division of Biology, California Institute of Technology, Pasadena, California, United States of America; Vanderbilt University, United States of America

## Abstract

Timing activity to match resource availability is a widely conserved ability in nature. Scheduled feeding of a limited amount of food induces increased activity prior to feeding time in animals as diverse as fish and rodents. Typically, food anticipatory activity (FAA) involves temporally restricting unlimited food access (RF) to several hours in the middle of the light cycle, which is a time of day when rodents are not normally active. We compared this model to calorie restriction (CR), giving the mice 60% of their normal daily calorie intake at the same time each day. Measurement of body temperature and home cage behaviors suggests that the RF and CR models are very similar but CR has the advantage of a clearly defined food intake and more stable mean body temperature. Using the CR model, we then attempted to verify the published result that orexin deletion diminishes food anticipatory activity (FAA) but observed little to no diminution in the response to CR and, surprisingly, that orexin KO mice are refractory to body weight loss on a CR diet. Next we tested the orexigenic neuropeptide Y (NPY) and ghrelin and the anorexigenic hormone, leptin, using mouse mutants. NPY deletion did not alter the behavior or physiological response to CR. Leptin deletion impaired FAA in terms of some activity measures, such as walking and rearing, but did not substantially diminish hanging behavior preceding feeding time, suggesting that leptin knockout mice do anticipate daily meal time but do not manifest the full spectrum of activities that typify FAA. Ghrelin knockout mice do not have impaired FAA on a CR diet. Collectively, these results suggest that the individual hormones and neuropepetides tested do not regulate FAA by acting individually but this does not rule out the possibility of their concerted action in mediating FAA.

## Introduction

Genetic screens have teased apart the molecular underpinnings of many circadian behaviors and processes. Many aspects of circadian rhythms such as sleep-wake cycles have a known genetic basis [1], [2], [3] as well as a neural substrate, the suprachiasmatic nucleus (SCN) of the hypothalamus [4]. Some other circadian-like phenomena such as increasing activity prior to scheduled meal delivery are not as well characterized at the neural and molecular levels [5].

Food anticipatory activity (FAA), which is the increased locomotion animals exhibit prior to receiving a daily scheduled meal, does not require an intact SCN in rats [6], hamsters [7] and mice [8]. In the genetic approaches to FAA, traditional circadian genes such as *Clock* [9], *Cry1/Cry2* [10], *NPAS2* [11], *Per2* [12], *and Bmal1* [13], [14] have yielded mixed results, suggesting that FAA may be regulated by non-traditional circadian controls and that feeding pathways would be worth pursuing to delineate circuitry. Since food intake is the obvious entry point for FAA, it is reasonable to observe mice with mutations influencing feeding behavior and/or body weight homeostasis. Recent studies using knockout mice for the hunger promoting hormone ghrelin and its receptor have suggested that ghrelin receptor neurons are required for the full expression of FAA [15], [16] but these results are not supported by a separate study [17]. Melanocortin-3 receptor mutant mice, which have increased adiposity, have impaired FAA [18] while the hyperphagic and obese 5HT2C knockout mice actually have enhanced FAA [19].

By reviewing the current feeding and FAA literature, we surmised that orexin, neuropeptide Y (NPY), ghrelin and leptin were either implicated directly in published reports or plausible targets that might regulate FAA [20], [21], [22], [23], [24], [25], [26], [27], [28], [29]. Prior studies have documented impairment in FAA of orexin null [30] or neuronally ablated mice [31] but the original study of orexinergic neurons and FAA in rats did not suggest a role in mediating FAA [32]. With respect to leptin, one study in the genetically obese Zucker rat found that leptin deficiency actually enhanced FAA [33]. However, though these results do give insight into the role of a specific neuropeptide in regulating FAA, differences between model organisms, strain backgrounds, activity measurements, and other methodological differences make direct comparisons between these strains difficult. Therefore we examined mice with single deletions of each gene in detail using video-based computer vision to assess the behavioral changes accompanying calorie restriction (CR) and the ability of these mutant mice to anticipate a daily scheduled meal.

## Results

### Comparison of home cage activity and body temperature during calorie or temporally restricted feeding

Many studies of FAA use a restricted feeding (RF) schedule where animals are given a window of time with *ad libitum* (AL) access to food and no access at all other times. Our studies using four single gene knock-outs (KOs) (described below) were done by performing CR where mice were given 60% of their AL food intake value at a specific time every day. To assess if RF and CR paradigms are similar or different, we performed a study to measure entrainment of body temperature and activity under both conditions.

**Figure 1:**
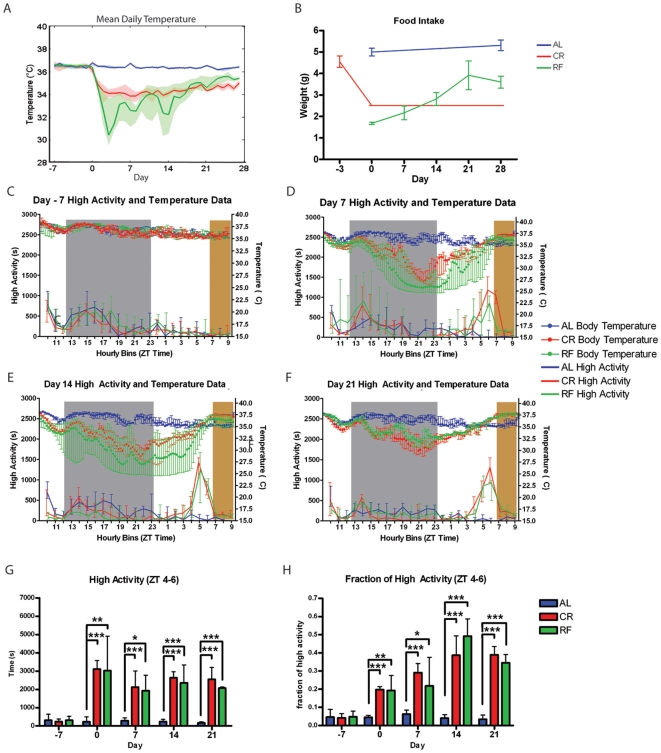
Comparison of behavior and body temperature under temporally and calorically restricted feeding conditions. (A) Mean daily temperature for each group of mice during the experiment. The bold line represents the median of the mean daily temperatures for individual mice, and the shaded regions represent the IQR. (B) Food intake for each group of mice. The line represents the mean and the bars represent the SEM. (C) Temperature and high activity seconds per hr seven days prior to beginning the special feeding regimen. Median temperature is represented by the filled circles in the top half of the panel (corresponding to the right y-axis) and median high activity is represented by the solid line in the bottom half of the panel (corresponding to the left y-axis). The bars represent IQR. (D) Temperature and high activity seconds per hourly bin seven days after the start of the special feeding regimen. (E) Temperature and high activity seconds per hourly bin 14 days after the start of the special feeding regimen. (F) Temperature and high activity seconds per hourly bin 21 days after the start of the special feeding regimen. (G) Seconds of high activity observed during the three hours prior to feeding (ZT 4–6) for the duration of the experiment. Medians and IQR are shown. (H) Seconds of high activity observed during the 3 hrs prior to feeding (ZT 4–6) divided by total seconds of high activity during the entire recording period. *  =  p<0.05, **  =  p<0.05, ***  =  p<0.001 (Kruskal-Wallis Test with Dunn's Post Test). n = 9 for AL, n = 8 for CR, and n = 6 for RF.

For mice fed AL, average core body temperature (the average temperature of each mouse across the entire day) remained between 35.9 and 37.2°C for all mice for the entire duration of the experiment. For mice on CR, the mean temperature was lowered, ranging from 33.9°Cto 34.6°C across individual mice (Fig. 1A). These mice had a significantly lower mean daily body temperature compared to day -1 (one day prior to starting CR) on days 2, 3, 6, 7, 8, 9, 10, 11, 13, 14, and 16 (p<0.05, Friedman's Test with Dunn's Post-Test). Mice on an RF schedule had a mean temperature for the entire 28 days of the special feeding schedule ranging from 32.0°Cto 34.9°C across individual mice. The mice on RF schedules had a significantly lower temperature from day 2 to day 9 and on days 13 and 14 of the experiment relative to their temperature on day -1 (Fig. 1A). After day 14, there is no significant difference between the mean daily temperature and that of the temperature before RF. The mean daily temperature of mice on CR and RF was significantly lower than that of mice on AL feeding all days of the experiment following day 0 of the special feeding schedule (p<0.001, Kruskal-Wallis Test). Although there were no significant differences in mean daily body temperature between mice on CR or RF (Kruskal-Wallis test), we noted that mice on an RF diet exhibited a much larger standard deviation in daily temperature, ranging from 1.35 to 2.32 degrees during days 3 to 14 of special feeding. In contrast, mice on a CR diet had a standard deviation in average daily temperature that remained between 0.39 and 0.62 during the same time period. Prior to special feeding, variation in the daily average temperature across individual mice was between 0.21 and 0.42 for the mice that were later placed on CR and between 0.18 and 0.37 for the mice that were later placed on RF, respectively.

Mice on an AL diet ate approximately 5 grams of food daily on average, as measured on days 0 and 28 of the experiment (Fig. 1B). On day 0, mice fed 60% of their normal food intake, 2.5 g, ate significantly more than those on RF, which only ate 1.6 grams (p<0.001, Mann-Whitney Test). There was no significant difference in food intake between mice on CR and those on RF on days 7 and 14. On day 21 and day 28, mice on RF ate significantly more than those on CR. Thus, mice on RF learned to eat more food as a function of time and experienced drastically different amounts of CR across the experiment: 31.5% CR on day 0, 39.14% on day 7, 56.33% CR on day 14, 78% on day 21 and 72% on day 28 (calculation was based on the AL average daily intake of 5 grams).

On day -7 (before any dietary change), there were no differences in behavior between mice on CR, mice on RF, and mice fed AL (Fig. 1C). Also, at this time all three cohorts had a similar core body temperature across the light:dark cycle. On day 7, mice on RF and CR exhibited significantly more high activity (defined as hanging, jumping, walking, and rearing) than mice fed AL during the hrs preceding meal time, ZT 4-6 (by convention ZT 12 is “lights off” as these mice are on a 13 hour [hr] light: 11 hr dark cycle) and significantly less activity at some points in the dark cycle, particularly the last several hrs of dark (ZT 21-23 for the RF and ZT 22 for the CR mice) (p<0.05, Kruskal-Wallis Test with Dunn's Post Test). There were no significant differences between mice on CR and RF at any of the twenty-four bins. After 7 days of modified feeding, RF and CR mice experienced a decrease in body temperature during the beginning of the dark cycle (around ZT 13). RF had a sharper decrease in body temperature relative to CR; however, both RF and CR reached nadir near 25°C. Most CR mice showed increased body temperature near ZT 22 while most RF mice had a delay to ZT 24, although the large interquartile ranges (IQR) indicate a large amount of variability in temperature across individuals. Both RF and CR groups reached peak body temperatures near 37.5°C by ZT 10 (Fig. 1D). On day 14, both CR and RF mice again showed significantly more activity during the several hrs preceding food delivery (p<0.05, Kruskal Wallis Test with Dunn's post test) (Fig. 1E). RF and CR mice experienced a decrease in body temperature during the beginning of the dark cycle (around ZT 13). Most RF mice exhibited a sharper decrease in body temperature relative to CR although it was less drastic than observed on day 7; however, both RF and CR reach nadir near 27.5°C. Both CR and RF reached peak body temperatures near 37.5°C by ZT 10 (Fig. 1E). On day 21, mice on RF had significantly more activity than those fed AL at bins ZT 3 and 5–8 (Fig. 1F). CR mice had significantly more activity than those fed AL from ZT 4 to 6, starting from three hrs prior to mealtime until three hrs after they are fed at ZT 7. On day 21, the AL control mice continued to maintain stable body temperatures while the RF and CR mice experienced a decrease in body temperature during the beginning of the dark cycle. Both RF and CR reach nadir near 30.0–32.0°C and began to increase body temperature near ZT 21. As with day 14, temperature reaches its peak during feeding.

When the total number of seconds of high activity is summed over the 3 hrs preceding feeding (ZT 4–6), mice on both CR and RF diets had significantly more high activity than the mice on AL diets from day 0 onwards (Fig. 1G). When the total seconds of high activity in the 3 hrs preceding feeding is normalized by dividing by the total number of seconds in the entire 24 hr recording period, both CR and RF mice had a significantly greater fraction of activity than mice on AL diets on days 0, 7, 14, 21, and 28 (Fig. 1H).

These results suggest that activity patterns between mice fed CR and RF are very similar, particularly after fourteen days of specialized feeding regimens. This corresponds to when mean daily temperature and food intake are very similar. During the earlier time point in the specialized feeding regimen (day 7), when mice on RF were eating less than those on CR, both temperature and activity were lower for mice on RF. At day 21, when both temperature and food intake has increased, mice on CR are slightly less active than those on RF, although CR mice and those on RF do not exhibit any significant difference in activity during the light cycle.

### Calorie restriction of orexin knockout mice

**Figure 2:**
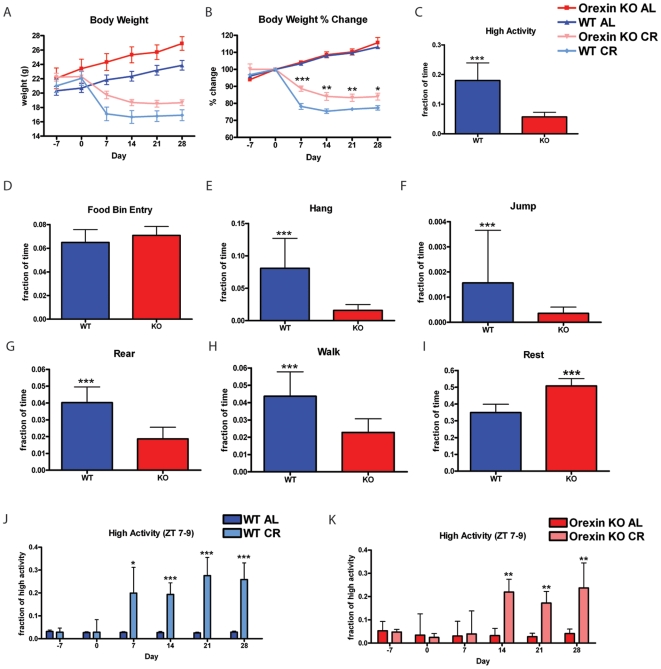
Calorie restriction of orexin knockout mice. (A) Body weights of orexin KO and WT mice on AL and CR feeding conditions. (B) Percent gain and loss of weight relative day 0 of the experiment. Orexin KO mice are resistant to weight loss when compared with WT CR mice (C) Fraction of time engaged in high activity behaviors (sum of hanging, jumping, rearing and walking) observed on day -7. (D) Fraction of time engaged in food bin entry, (E) fraction of time engaged in hanging, (F) fraction of time engaged in jumping, (G) fraction of time spent rearing, (H) fraction of time spent walking, and (I) fraction of behavior of spent in a resting state on day -7 of the experiment. n = 12 orexin KO and n = 23 orexin WT. (J) High activity behaviors during the 3 hrs prior to scheduled meal delivery (ZT 7–9) for WT mice on AL (n = 18) and CR (n = 9) and (K) Orexin KO mice on AL and CR (n = 6 for both groups). Statistics were performed using Mann-Whitney Test *  =  p<0.05; **  =  p<0.01; ***  =  p<0.001. Error bars represent the IQR for behavioral data and SEM for body weight data.

Under AL feeding conditions, both C57BL/6 orexin KO mice and WT littermate controls gained weight (Fig. 2A). Under the CR feeding conditions (beginning on day 0), orexin KO mice lost weight but not as severely as the WT controls (Fig. 2A). Food intake for orexin WT mice was 4.91±0.8 and 4.81±1.0 for orexin KO mice (mean and SD). To examine the body weight changes more carefully, we normalized the data by dividing the weight at each measurement by the weight on day 0 prior to the start of CR and observed that orexin KO and WT mice had similar percent weight gains when on AL feeding conditions (Fig. 2B). However, on a CR diet, orexin KO mice appeared refractory to weight loss on CR, as orexin KO mice on CR maintained a significantly higher percentage of their starting body weight than WT controls on CR feeding conditions (P<0.05, Mann-Whitney) (Fig. 2B).

We next examined home cage activity on day -7 of the experiment to determine if orexin KO mice had similar behavior to controls. Orexin KO mice show significant hypoactivity compared to their WT littermates for high activity behaviors (P<0.001, Mann-Whitney) (Fig. 2C). Food bin entry (Fig. 2D) was not different between orexin KO and WT mice. Hanging (Fig. 2E), jumping (Fig. 2F) rearing (Fig. 2G), and walking (Fig. 2H) showed a statistically significant decrease in orexin KO mice (P<0.001, Mann-Whitney). Consistent with less high activity behavior, the orexin KO mice spent a significantly larger fraction of total time in a resting state (defined as >30 seconds of immobility) (Fig. 2I).

The fraction of high activity behaviors observed during the 3 hrs preceding feeding time, which for these mice and all subsequent transgenic studies was ZT 7–9 as feeding occurred at the beginning of ZT 10, relative to the total high activity was not significantly different between WT mice on AL and CR on days −7 and 0. Beginning on day 7, WT CR mice showed a significant increase in high activity relative to WT AL (P<0.05, Mann-Whitney), which increased in significance in subsequent days (P<0.001, Mann-Whitney) (Fig. 2J). Littermate orexin KO mice were assayed in the same manner and on days −7, 0, and 7 there was no observable difference between orexin KOs on AL or CR (Fig. 2K). Orexin KO on CR did not show significant elevation in high activity preceding meal time until day 14 (Fig. 2K). Since the increase in FAA was slower to develop in orexin KO mice but reaches a similar magnitude (20–25% of total high activity occurs in ZT 7–9), this suggests that orexin KO mice have delayed acquisition of but no impairment in FAA.

### Calorie restriction of leptin knockout mice

**Figure 3 pone-0018377-g003:**
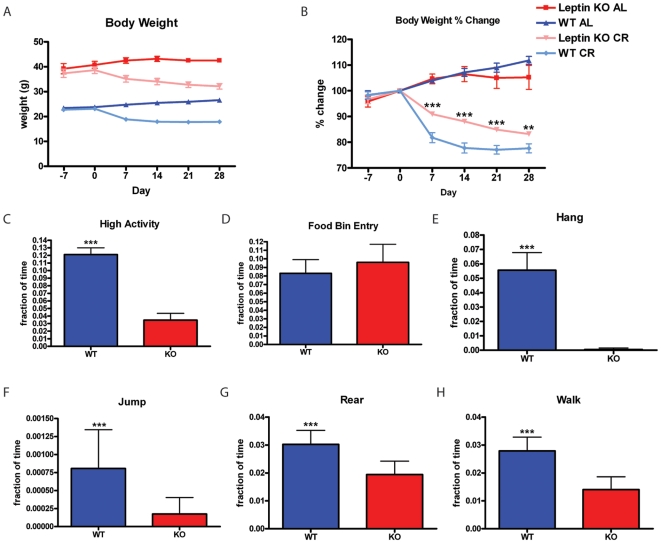
Calorie restriction and baseline home cage behavior of leptin knockout mice. (A) Leptin KO and WT mice on AL feeding conditions gain weight while Leptin KO and WT mice on CR lose weight. (B) Leptin KO mice on AL and CR feeding conditions have similar percent gain and loss of weight relative to percent changes in weight of WT controls on AL and CR feeding conditions. Leptin KO mice on CR are more resistant to weight loss (p<0.01 for Leptin KO CR vs. WT CR). (C) High activity behaviors of WT mice are much higher than Leptin KO mice. WT and Leptin KO mice have comparable (D) food bin entry on AL feeding. WT mice (E) hang, (F) jump, (G) rear and (H) walk more than Leptin KO mice. Statistics were performed using the Mann-Whitney Test *  =  p<0.05; **  =  p<0.01; ***  =  p<0.001. Error bars represent IQR for behavioral data and SEM for body weight data. n = 16 WT AL and n = 15 KO AL.

Leptin KO mice have an obesity phenotype that must be taken into account when interpreting data from leptin KO and WT controls as the leptin KO mice began the experiment weighing almost double that of control weight (Fig. 3A). Food intake for leptin WT mice was 3.9±0.8 grams and for leptin KO mice food intake was only 3.6±0.7 grams per day. Under AL feeding conditions, both C57BL/6J leptin KO mice and WT controls gained weight throughout the period of the experiment. Under the CR feeding conditions (beginning on day 0), both leptin KO mice and WT controls lost weight throughout the period of CR feeding (days 0 through 28) (Fig. 3A). Leptin KO and WT mice on AL feeding conditions had similar percent weight gain; however, percent weight loss of leptin KO and WT mice on CR is statistically different. Leptin KO mice on CR lost weight at a slower rate (Fig. 3B). Overall the high activity behaviors of WT mice were much higher than leptin KO mice on AL feeding (day -7) where WT mice spent about 12% of their total time engaging in hanging, jumping, rearing, and walking; whilst leptin KO spent only 3% of their total time engaged in such behaviors (Fig. 3C), consistent with prior reports [34]. However, the amount of food bin entry displayed by leptin KO mice was indistinguishable from that of controls (Fig. 3D). The most significant difference in an individual high activity was in hanging behavior, where leptin KO mice barely hung at all, but control mice spent 5% of their total time hanging (Fig. 3E). A similar but less dramatic reduction in the amount of time spent jumping, rearing, and walking was observed in leptin KO mice as well (Fig. 3F–H).

**Figure 4 pone-0018377-g004:**
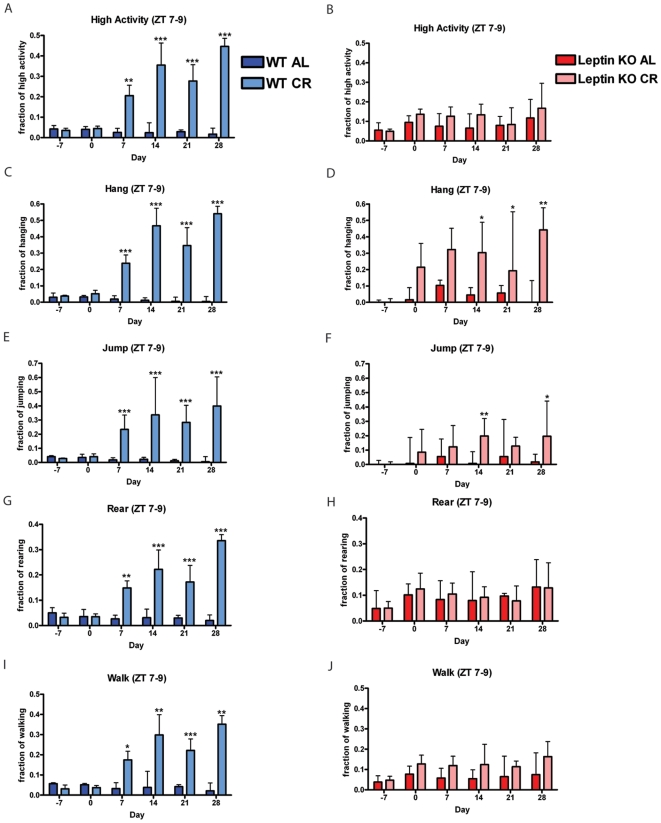
Calorie restriction of leptin knockout mice. Measure of fraction of time engaged in activity during the anticipatory period prior to feeding (ZT 7–9). Beginning from day 7, WT AL and WT CR mice have statistically significant differences in fraction of time spent engaging in (A) total high activity behaviors (sum of hang, jump rear, and walk) as well as individual behaviors such as (C) hang, (E), jump, (G) rear, and (I) walk. In contrast, Leptin KO mice on AL and CR feeding have no statistical difference in (B) total high activity behaviors. (D) Hang reaches statistical significance at day 14 that is sustained through day 28 and (F) jump reaches transient statistical difference at day 14 and 28. (H) Rear and (J) walk is not statistically different between AL and CR. Statistics were performed using the Mann-Whitney Test with post-test, *  =  p<0.05; **  =  p<0.01; and ***  =  p<0.001. Error bars represent IQR. n = 8 WT AL; n = 8 WT CR; n = 7 KO AL and n = 8 KO CR.

We next examined the normalized high activity during the anticipatory time ZT 7–9 measured every 7^th^ day for 28 days. For WT mice on AL and CR, the fraction of high activity on day 0 when the WT CR group is switched from AL to CR feeding was similar (Fig. 4A). Beginning on day 7, WT CR had a significant increase in fraction of high activity preceding feeding which remained highly significant through day 28. Of the high activity behaviors, 20–50% occurred in ZT 7–9 for WT CR whereas control values were typically below 10%.

Interestingly, leptin KO AL mice showed no statistically significant difference in fraction of high activity relative to the leptin KO CR group (Fig. 4B). Leptin KO mice on an AL diet have a larger fraction of their total activity in ZT 7–9 than WT control mice on AL diets— a median of almost 10% of the total high activity for AL leptin KO mice occurred during ZT 7–9 from day 0 onwards in contrast to less than 5% for AL controls at all time points (Figure 4A–B). Beginning on day 7, WT CR had a significant increase in hanging behavior with a trend continuing to day 28 (Fig. 4C). On day -7, leptin KO AL and CR (both groups on AL feeding) had a negligible fraction of hanging. On days 0 (beginning of CR) and 7, leptin KO CR experienced a trend of increased hanging (Fig. 4D). Starting on day 14 of the experiment leptin KO CR group showed a statistically significant greater fraction of hanging behavior relative to the leptin KO AL control (Fig. 4D). Jumping behavior during the three hours preceding feeding showed a similar but less pronounced trend as did hanging with significant increases occurring on days 14 and 28 only (Fig. 4F). WT CR mice conducted at least 15% of their jumping behavior during ZT 7–9 from days 7 through 28 where this behavior was significantly increased over WT AL values (Fig. 4E).

As expected, on days -7 and 0, WT mice on AL and CR had a similar fraction of rearing and walking during ZT 7–9 (Fig. 4G,I). Beginning on day 7, WT CR showed a significant increase for both rearing and walking that persisted for the duration of the experiment. In contrast, leptin KO mice did not increase rearing and walking in anticipation of mealtime appreciably on a CR diet (Fig. 4H and 4J, respectively).

### Calorie restriction of neuropeptide Y knockout mice

**Figure 5 pone-0018377-g005:**
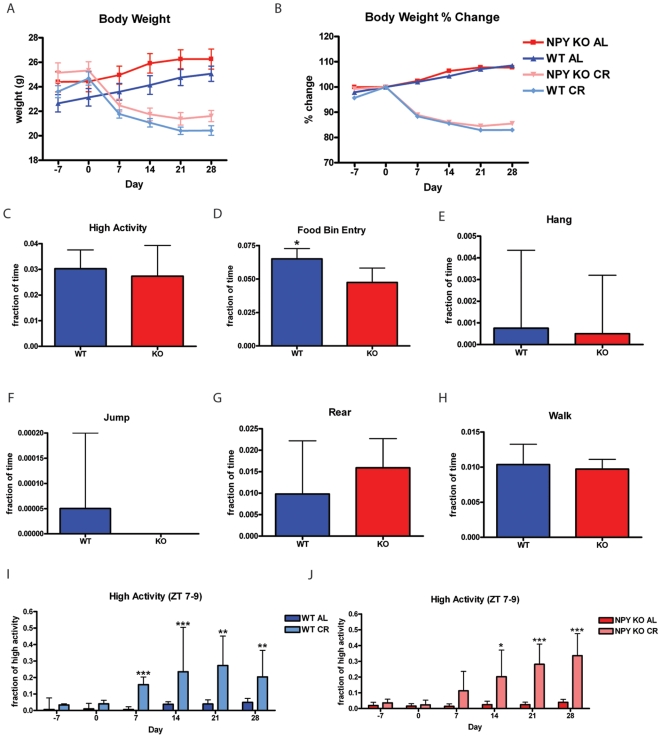
Calorie restriction of NPY knockout mice. (A) Body weights of NPY KO and WT mice on AL and CR feeding conditions. (B) Percent gain and loss of weight relative to day 0. (C) High activity behaviors of NPY KO and WT mice on day -7. (D) Food bin entry (E) hang, (F) jump, (G) rear and (H) walk on day -7. Statistics were performed using the Mann-Whitney Test with post-test. Error bars represent IQRs. n = 16 WT and n = 18 KO. (I) Normalized high activity behaviors in the 3 hrs preceding feeding for WT CR and WT AL. (J) Normalized high activity behaviors in the 3 hrs preceding feeding for NPY KO CR and WT AL. Statistics were performed using the Mann-Whitney Test, *  =  p<0.05; **  =  p<0.01; ***  =  p<0.001. Error bars represent IQR. n = 8 for all groups at all time points in panels I and J.

Under AL feeding conditions, both 129SvJ NPY KO mice and WT controls gained weight (Fig. 5A–B). Under the CR feeding conditions, both NPY KO and WT controls lose a similar percent of weight (Fig. 5A–B). There was no difference in baseline high activity behaviors between NPY KO and WT mice on AL feeding (day -7) (Fig. 5C); however, the amount of food bin entry displayed by WT mice was significantly higher than for NPY KOs (Fig. 5D). Despite this difference in food bin entry, the food intake values for WT and NPY KO on AL were nearly identical, with WT mice consuming 5.7±2.3 grams and NPY KO mice consuming 4.7±0.8 grams of chow per day on average. We examined the individual high activity behaviors and saw that there were no significant differences in hanging (Fig. 5E), jumping (Fig. 5F), rearing (Fig. 5G) and walking (Fig. 5H). WT mice on CR began showing a statistically significant increase in the fraction of high activity during ZT 7–9 relative to AL controls starting on day 7 and onwards (Fig. 5I). NPY KO mice on a CR diet noticeably increased activity beginning on day 7 and began showing a statistically significant increase on day 14 onward (Fig. 5J). The lack of significant increase in FAA in NPY KO mice until day 14 could be interpreted as a delayed onset of FAA in NPY KO mice since FAA is significantly increased in NPY WT mice on CR from day 7 though it is clear that there is a strong trend toward increased activity in the three hrs preceding feeding at day 7 in the NPY KO mice on CR.

### Calorie restriction of ghrelin knockout mice

**Figure 6:**
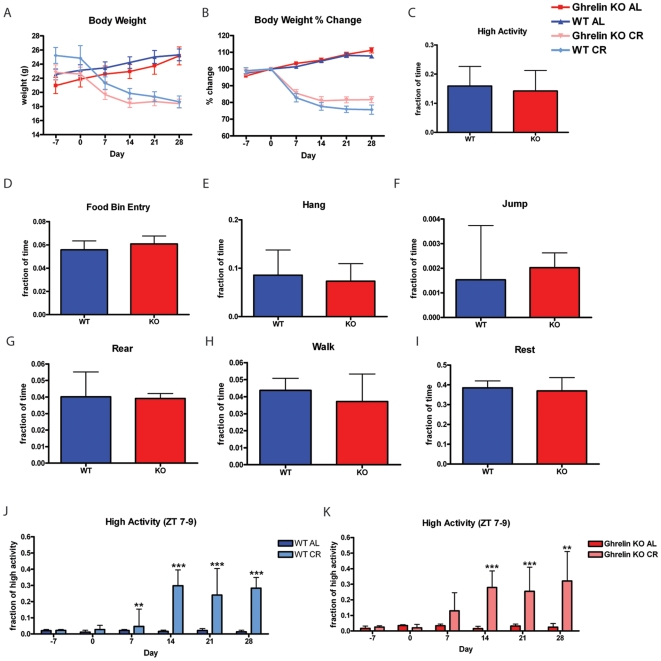
Calorie restriction of ghrelin knockout mice. (A) Body weights of Ghrelin KO and WT mice on AL and CR feeding conditions. (B) Percent gain and loss of weight relative to day 0. There is no measurable difference in (C) High activity behaviors of Ghrelin KO and WT on day -7. (D) Food bin entry (E) Hang, (F) Jump, (G) Rear, (H) Walk, and (I) Sleep behaviors are not different among WT and KO groups on day -7. n = 16 for panels C–I. (J) Normalized high activity behaviors in the 3 hours preceding feeding for WT AL and WT CR. (K) Normalized high activity behaviors in the 3 hrs preceding feeding for KO AL and KO CR mice. Statistics were performed using the Mann-Whitney Test with post-test, *  =  p<0.05; **  =  p<0.01; and ***  =  p<0.001. Error bars represent IQR. n = 8–9 for panels J and K.

Under AL feeding conditions, both ghrelin KO mice and WT controls gained weight (Fig. 6A,B). Under CR feeding schedules, both ghrelin KO and WT controls lose about 20% of their body weight by the end of the experiment (Fig. 6A–B). Behavior profiles of ghrelin KO and WT mice one week prior to the start of feeding schedules show no measurable behavioral differences in terms of food bin entry, hanging, jumping, rearing, walking, and resting (Fig. 6C–I). Food intake was 4.4±0.7 for ghrelin KO and 5.1±1.26 grams for ghrelin WT controls. By day 7 of calorie restriction, WT mice begin showing a small but statistically significant increase in the fraction of high activity relative to AL controls (Fig. 6J). Ghrelin KO mice also trend toward an increase in the fraction of high activity by day 7; however, this difference is not statistically significant (Fig. 6K). From day 14 onward, both ghrelin KO and WT mice show a robust increase in the fraction of high activity in the three hours before scheduled meal time. The lack of a significant increase in FAA in ghrelin KO mice on day 7 could be interpreted as delayed onset of the acquisition of FAA but in contrast with orexin KO mice on CR diets there is an obvious trend toward increasing high activity behaviors in NPY KO CR mice at this time point.

## Discussion

The similarity between the RF and CR models was evident from core body temperature oscillations, weight change, and home cage activity. Though the RF mice did show increased core body temperature as time progressed, the fluctuations in temperature during the first few days of the RF schedule and large variation across individuals were much more extreme than for CR; the initial minimal but gradually increasing food intake suggests that RF mice are learning that they have a short temporal window in which to eat. While AL mice had a constant food intake between 5.0 and 5.5 g a day, RF mice showed an increase in mean daily food intake from 1.8 grams on day 0 (first day of RF) to a peak of 4.0 grams at day 21 (Fig. 1B). Because RF mice have a greater magnitude of temperature variability as well as variability in food intake whereas mice on CR had a more stable mean daily temperature and fixed food intake, we advocate the use of CR over RF in assaying for FAA. In addition, CR only requires a single entry into the mouse room whereas RF requires an entry to deliver and another entry to take away food, so CR is less disturbing to the mice as well as less labor-intensive. In terms of food anticipatory behavior, RF and CR mice exhibited similar changes but the CR group was the first to achieve statistically significant increased FAA. Thus, as with temperature, CR mice behave similarly to RF mice but with less variability.

Two previous studies concluded that orexin is necessary for the full expression of FAA, based on 4 hrs of restricted feeding over the course of nine days [30], [31]. The study by Akiyama et al. showed that orexin/ataxin-3 transgenic mice, which are ablated for orexinergic neurons, have blunted FAA relative to controls [31]. Orexin/ataxin-3 actogram data suggested that these mice have a delayed entrainment; however, in the 2 hr window prior to feeding they do show an increase in activity even though this is significantly lower than the activity of WT counterpart mice [31]. In a follow-up to this study using the same experimental conditions for inducing FAA, Kaur and colleagues tested whether it is the orexin neuropeptide (as opposed to orexinergic neurons which were ablated in the study by Akiyama et al) that is necessary for FAA [30]. Surprisingly, the authors are able to observe FAA in WT controls on RF feeding within 1–3 days of RF and over the next several days they did not see appreciable anticipatory activity in orexin KO mice [30]. Although much of Akiyama et al. and Kaur et al. data is consistent with orexin mediating FAA, the 1) brief duration of their food restriction experiments, 2) lacking a parallel group of orexin ablated/null AL controls, and 3) not correcting/normalizing for differences in global activity caused by orexin ablation/deletion call into question the significance of these findings.

In fact, the first study of orexin and FAA, performed by lesioning orexinergic neurons using saporin conjugated neurotoxin, showed that orexin neuron-depleted rats had robust FAA [32], consistent with what we have observed. Another recent study of orexin KO mice [21], although focused on the role of orexin in narcolepsy, also suggests the presence of essentially normal FAA in these mice. Thus, while orexin neurons have a clear role in sleep and narcolepsy, its function in FAA is both unclear and likely to be indirect. We made an interesting observation that orexin KOs lost weight less rapidly than wild type mice; this may be related to the reduced activity of KO mice when on AL feeding conditions and the delayed onset of statistically significant anticipatory activity or it could be due to altered body weight homeostasis in orexin KO mice. In contrast to the short duration of the aforementioned studies, our experiment with orexin KO mice utilized CR to induce anticipatory activity and continued for 28 days of CR. In our experience, it is important to distinguish between hunger induced hyperactivity, which is quite generalized and does not occur only in the time before what will become daily meal time, and FAA which is specific to the few hrs before daily feeding time. Normalizing the data by dividing the activity observed in the hours preceding feeding by the total activity is an important way to address this concern.

Resistance to weight loss of leptin KO mice is consistent with previous studies documenting this phenomenon [35], [36]. Comparing individual high activity behaviors for leptin KO mice versus WT controls before scheduled feeding demonstrates that leptin KO mice were severely hypoactive, consistent with other studies [34]. Food bin entry time (Fig. 3D) and food intake values were both similar between leptin KO and WT mice, showing that at the start of our experiment (when leptin knockout mice are already overweight) the leptin KO mice were not hyperphagic. Because leptin KO mice are hypoactive and have modified physiology, it is imperative to compare leptin KO mice on CR and AL in parallel. Leptin KO mice on CR had an interesting behavioral response in that some high activity behaviors such as walking and rearing did not increase appreciably while hanging and jumping actually did show significant increases after two weeks of the CR feeding schedule when they have reach about 90% of their initial body weight. This suggests that leptin KO mice do have some ability to sense meal timing but do not express all behaviors typically associated with FAA in our studies. Since walking and rearing are much more common behaviors than hanging and jumping, they dominate the measurement that we term “high activity” and it appears falsely that there was no FAA in leptin KO mice. The lack of full expression of FAA in leptin KO mice could be due to the action of leptin receptor expressing neurons promoting increased walking and rearing, but not hanging and jumping, during the hours preceding mealtime or be a more indirect effect of their altered metabolism and behavior caused by leptin deletion. One prior study by Mistlberger et al. showed that genetically obese Zucker rats (containing a point mutation that inactivated the leptin receptor [37], [38], [39]) had enhanced FAA when kept on 22 days of RF with a 3 hr RF window [29]. Another study using rats showed that chronic leptin treatment decreased running wheel activity prior to feeding time on an RF regimen [40]. Thus, it may be worth investigating the contribution of leptin receptor neurons to FAA. Future experiments should utilize genetic tools to delete leptin receptor expression in specific populations of neurons as was done by Bradford Lowell, Joel Elmquist, and colleagues to delineate the crucial population of leptin receptor expressing neurons responsible for mediating body weight homeostasis [24], [25], [41], [42].

Since the loss of the anorexigenic hormone leptin decreased some aspects of FAA we next tested the well established orexigenic compound neuropeptide NPY. Given its expression in the arcuate nucleus of the hypothalamus, NPY is poised to control FAA, leading us to investigate the phenotype of NPY KO mice on CR [20], [43], [44], [45], [46], [47], [48], [49]. In our studies of NPY KO mice on CR, where we observed that the NPY KO CR mice formally have a slight delay in the acquisition of FAA but not in the ultimate expression of FAA, it is important to note that the control mice were not littermates of the NPY KO mice as both groups of mice were purchased from Jackson Laboratories and were derived from separate breeding colonies. Thus, given this caveat and the strong trend toward increased activity in the NPY KO CR mice at day 7, we feel that it would not be correct to conclude that NPY KO mice have a true delay in acquisition of FAA unless this result was repeated with littermate controls.

The ghrelin ligand could conceivably activate NPY/AgRP neurons in the arcuate nucleus to mediate FAA [50]. Deletion of the ghrelin ligand in our study did not alter any aspect of body weight homeostasis, activity behaviors, or the amount of FAA in response to 60% CR. At least two studies of the ghrelin receptor KO mouse suggest that ghrelin receptor expression is required for the full expression of FAA [15], [16] and one additional study demonstrates a role for ghrelin in CR induced hyperactivity [51] but one other published study of the ghrelin ligand KO did not detect an impairment of FAA in the ghrelin ligand KO mice [17]. This opens up the possibility that ghrelin itself is not the ligand mediating receptor activation to promote food anticipatory activity and that there is another ligand of the ghrelin receptor that mediates the full expression of FAA.

In conclusion, we made a careful comparison of FAA in several strains of mutant mice with known defects in feeding, arousal behavior, or acquisition and/or expression of FAA. We took care to standardize for physiological and behavioral differences between WT and KO mice and also to study FAA for a long duration (at least 28 days). Overall, our results do not support critical roles for any of the genes studied—leptin, orexin, NPY, and ghrelin—acting individually in mediating FAA by CR, helping to clarify the food anticipation literature and facilitate the investigation of other candidate genes/pathways or combinatorial approaches that will concomitantly eliminate multiple pathways.

## Materials and Methods

### Mouse strains and husbandry conditions

All experiments were approved by the Caltech Animal Care and Use Committee. All mice were allowed AL access to LabDiet Laboratory Rodent Diet 5001 and water and were on a 13 hrs of light 11 hrs of dark cycle. For designating time, ZT 12 was designated as the commencement of lights-off. Feedings for Figure 1 occurred at ZT 7 and for Figures 2–6 feedings occurred at ZT 10. AL controls received a food pellet of arbitrary size at the same time as CR feedings to control for disturbance to sleep-wake cycles caused by feeding events. To genotype mice, DNA was obtained from tail clippings which were digested with proteinase K and the DNA was purified using an isopropanol precipitation. For genotyping the orexin genomic locus we used the following primers: orexin forward primer GACGACGCCTCAGACCTCCTGGG, orexin reverse primer TCACCCCCTTGGGATAGCCCTTCC and CCGCTATCAGGACATAGCGTTGGC for amplifying neomycin in the knockout allele. Genotyping primers for identifying ghrelin genotype by PCR were as follows: GAGTCTCCATCCCAAGAGGT and GTCTCCTGCTTCCCAGTTTA for identifying the WT allele and the following primers for amplification of LacZ which is part of the knockout allele: ACCATTTTCAATCCGCACCTC and GGTCAATCCGCCGTTTGTTC.

Orexin KO mice were kindly provided by Masashi Yanagisawa (UT Southwestern) and were on a C57BL/6J background and re-derived at the Caltech transgenic facility by crossing for an additional generation to C57BL/6. All orexin KO and control mice used in the study were derived from the same parents by intercrossing mice that were heterozygous for the orexin deletion. Several of the orexin mice used as “WT” controls were +/− for the orexin deletion (having one copy of the orexin gene deleted did not appear to have any effect on their behavior).NPY and leptin mice were provided directly by Jackson Laboratory (Bar Harbor, ME) and their website indicates primers and PCR conditions. Male leptin KO mice ob/ob (strain name: B6.V-Lep^ob^/J; stock number: 000632) were obtained from Jackson Labs on a C57BL/6J background. Leptin KOs and WTs were not littermates but were age and gender matched. Male NPY KO mice were obtained from Jackson Labs as knockouts (Strain Name: 129S-Npy^tm1Rpa^/J; stock number: 004545) on a 129S1 background and controls 129S1/SvIMJ (stock number: 002448) were purchased from Jackson labs. Ghrelin KO mice were kindly provided by Tamas Horvath with permission of Mark Sleeman on a mixed background of C57BL/6 and SJL/J and crossed for one to two generations onto C57BL/6J upon arrival and ovarian transfer rederviation at the Caltech animal facility.

### Temperature monitoring experiment

Twenty-six male C57BL/6J mice were singly housed for several days then surgically implanted with iButtons (Maxim Integrated Products) [52], which were programmed to record core body temperature rounded to the nearest 0.0625°C at 15 minute intervals for a period of 35 days (day -7 to 28). Mice were given at least seven days to recover, after which they were recorded for their first time point (Day -7) when all mice had free access to food. Starting on day 0, the mice were subdivided into three groups: calorie-restricted (n = 8), RF (n = 6), and *AL* (n = 9). Initially, the RF group contained eight mice; however, two died within the first two weeks of RF and were thus excluded from the study. Mice on CR and RF diets received automatic feeders which deposited either 2.5 g (60% of *AL* food intake) or an unlimited amount of food, respectively, at ZT 6 each day. At ZT 9, all three groups were disturbed, and food is removed from the cages of the RF mice. Mice were recorded for 23.5 to 24 hrs on day 0, 7, 14, 21, and 28 of their special feeding treatment, although Day 28 was excluded due to technical difficulties with the cameras. After day 30, mice were sacrificed to obtain the implanted iButtons.

### Behavioral Measurements

The videos of singly housed mice in the home cage were analyzed by an automated behavior recognition system, HomeCageScan 3.0 [53], [54], and data was output into twenty four one hr bins to facilitate understanding of the temporal structure of activity. Dim red lighting was provided during the 11 hr dark cycle by Philips 25 watt “party and deco” bulbs later replaced with red LEDs from LEDwholesalers.com. Home cage behavior measurement of orexin, leptin and NPY knockout mice began (day -7) at 9–10 weeks of age. Home cage behavior measurements were obtained by video recording mice from a perpendicular angle in their home cages and analyzing these videos using HomeCageScan software, which annotates for the following behaviors: remain low, pause, twitch, awaken, distance traveled, turn, sniff, groom, food bin entry, chew, drink, stretch, unassigned behaviors, hanging, jumping rearing, and walking. The sum of hanging, jumping, rearing, and walking are designated as high activity behaviors. Body weights of mice were weighed every 7^th^ day beginning from day −7 to day 28. Food intake was calculated for all mice prior to starting CR. Food intake was measured by placing approximately 50 grams of standard mouse chow in the food bin and measuring remaining chow mass 48 hrs later. Daily averages were computed per cohort and 60% of the daily average was the designated CR value. Extremely high values for food intake (due to food “grinding”) were excluded when calculating food intake and CR values.

### Statistical Analysis

Statistical significance tests were conducted using GraphPad Instat and previously described MATLAB code [53], [54]. For within group comparisons of temperature data we used Friedman's Test with Dunn's Post Test (eg, comparing the temperature of CR mouse to baseline values). As behavioral data did not follow a normal distribution, we used nonparametric tests, Mann-Whitney for comparing two groups and Kruskal-Wallis with Dunn's post-test for comparing three groups.
